# A Framework for Comparing Mouse Neoantigen Immunogenicity

**DOI:** 10.64898/2026.02.10.704454

**Published:** 2026-02-12

**Authors:** Peter J. Matulich, Carly N. Sprague, Victoria P. Schuster, Alyssa M. Granados, Rohan B. Chaudhari, Megan L. Burger

**Affiliations:** 1 Department of Cell, Developmental and Cancer Biology, Oregon Health and Science University, Portland, OR, USA; 2 Knight Cancer Institute, Oregon Health and Science University, Portland, OR, USA

**Keywords:** Neoantigen, antigen strength, peptide-MHC stability, preclinical tumor models, immunotherapy

## Abstract

Cytotoxic CD8+ T cell responses targeting tumor neoantigens are critical for immunotherapy efficacy and are widely studied across different preclinical mouse tumor models. Defined neoantigens are commonly introduced to enable tracking of tumor-specific T cells; however, variation in neoantigen choice may yield immune phenotypes attributable to differences in neoantigen immunogenicity, complicating interpretation of tumor-intrinsic mechanisms. Here, we determined the relative immunogenicity of a set of 25 commonly used mouse tumor-derived and model neoantigens to facilitate comparison of neoantigens across studies. We found that *in silico* predicted major histocompatibility complex (MHC) binding affinity poorly stratified *in vivo* immunogenicity. In contrast, experimental measurement of peptide-MHC complex stability (K_off_), more so than measured affinity (K_D_), closely correlated with the relative magnitude of neoantigen-targeted vaccine responses *in vivo*. Thus, we report the relative stability of a known set of commonly used neoantigens as a reference and provide a simple method to benchmark novel neoantigens against this library. This framework will allow contextualization of the level of immunogenicity of newly identified neoantigens and aid in comparative interpretation of tumor-immune phenotypes across studies.

## INTRODUCTION

Immunotherapy has dramatically reshaped the treatment of many cancers, particularly in the metastatic setting. Immune checkpoint blockade (ICB) has had the most success in the clinic, leading to remarkable improvements in patient outcomes and fueling a surge of research into cancer immunology and immunotherapy^1^. T cells are key mediators of ICB response and recognize mutated proteins in tumor cells as foreign. Only a subset of these mutated proteins are capable of generating an immune response and are known as “neoantigens”^2-4^. A new generation of immunotherapies including personalized cancer vaccines and adoptive T cell therapies are being developed that rely on identifying and targeting specific neoantigens^5^. This underscores the importance of studying neoantigen-specific T cell responses, including which mutations drive these responses and which can best be leveraged therapeutically.

While studying neoantigen-specific T cells is clearly a priority, identifying them in patient samples presents significant challenges. Neoantigens are unique to each patient requiring the sequencing of individual tumor samples and screening for potentially immunogenic mutations. To validate potential neoantigens, tumor-infiltrating lymphocytes must be directly screened for neoantigen-reactivity^2-4^. Therefore, to study neoantigen-specific immune responses, investigators have primarily turned to mouse models expressing defined neoantigens, which have been invaluable tools for understanding tumor-immune interactions. Typically, tumor-specific CD8+ or CD4+ T cell responses are studied by either expressing a model neoantigen (e.g., commonly used SIINFEKL from chicken ovalbumin) in a tumor or using a tumor line with known neoantigens (e.g., MC38 colorectal cancer). The T cell response to these neoantigens are then evaluated by monitoring either the endogenous T cell response or transferred T cell receptor (TCR) transgenic T cells with a cognate neoantigen-specific TCR. Unsurprisingly, it is becoming increasingly clear that the identity and context of neoantigen expression can significantly influence the corresponding anti-tumor T cell response^6,7^. Given that neoantigens have varying intrinsic immunogenicity, determining the contribution of tumor-intrinsic factors in regulating T cell responses is challenging when different neoantigens are expressed across different tumor systems. Therefore, it is important to develop an understanding of how to compare neoantigen immunogenicity across studies to disentangle neoantigen-intrinsic versus tumor-intrinsic phenotypes.

Several factors are known to influence neoantigen immunogenicity, with the primary determinant being the density of peptide-MHC (pMHC) complexes at the cell surface^8^. Because empty MHC molecules are unstable, a neoantigen’s ability to load onto MHC and remain bound is critical for determining its surface density. Antigen loading is tied to binding affinity, while the persistence of the pMHC complex at the cell surface is governed by its stability^9,10^. *In silico* algorithms have been developed to predict immunogenic peptides and their binding affinity^11,12^. These algorithms are trained on large datasets of experimentally measured pMHC affinities and large-scale peptide elution studies in which MHC-bound peptides are identified by mass spectrometry. As these algorithms are trained in part on binding affinity, they can report a predicted binding affinity for antigens. However, it remains unclear how effectively current prediction algorithms can discern relative immunogenicity of mouse neoantigens and whether these algorithms can confidently be used to compare neoantigens across studies, representing a key gap that we aim to address.

In this study, we selected 19 mouse tumor-derived neoantigens^13-20^ and 6 model neoantigens^21,22^ from various tumor studies and evaluated the concordance of *in silico* prediction outcomes with experimental measures of relative pMHC binding strength. We further assessed alignment of both *in silico* and experimental measures with ground-truth immunogenicity in an *in vivo* vaccine setting. We found that *in silico* predictions and, surprisingly, experimental pMHC binding affinity measurements poorly ranked the *in vivo* immunogenicity of the neoantigens; however, experimental pMHC stability measurements were highly correlated, consistent with accumulating evidence supporting strong predictive value of stability in other studies. These findings suggest that *in silico* predictions are unreliable for determining relative neoantigen immunogenicity and nominate experimental measurement of pMHC stability as a better metric. This work defines the relative pMHC stability of a set of commonly used mouse neoantigens as a reference for the field and provides a straightforward framework for benchmarking novel neoantigens against this reference to aid in comparison of neoantigen-driven immunity across tumor models.

## RESULTS

### In Silico Algorithms Similarly Rank the MHC I Binding Affinity of Neoantigens

**Figure 1.**
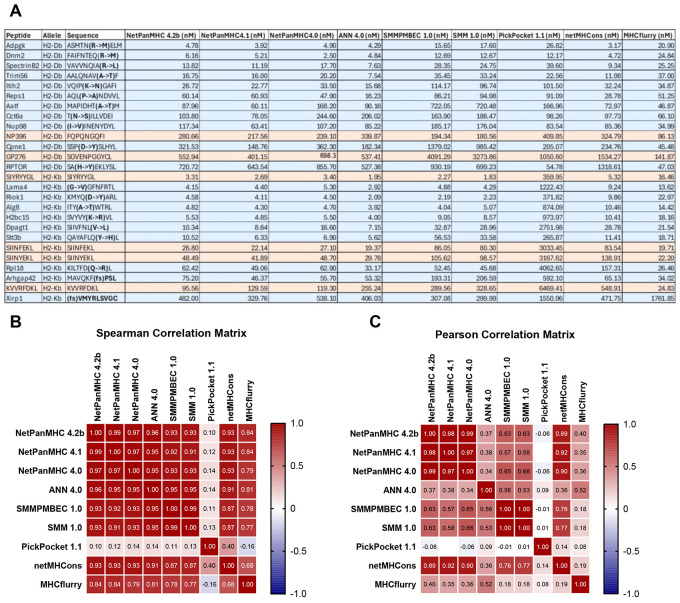
In Silico Algorithms Similarly Rank the MHC I Binding Affinity of Neoantigens (A) Table of mouse tumor-derived neoantigens (blue) with annotated wild-type versus mutated sequences and model neoantigens (orange) including allelic restriction (H2-D^b^ or H2-K^b^) and predicted IC50 values from the *in silico* algorithms indicated. (B-C) Correlation matrix of the predicted IC50 values for the neoantigen library in (A) between different *in silico* algorithms using the Spearman correlation coefficient (B) or Pearson correlation coefficient (C).

We sought to computationally predict and experimentally determine the relative immunogenicity of a library of 25 CD8+ T cell-reactive neoantigens. We selected 19 mouse tumor-derived neoantigens from seven different tumor systems and added six commonly used model neoantigens (Fig. 1A). We first inputted the assembled neoantigen peptide sequences through 9 publicly available *in silico* prediction algorithms that support the mouse MHC-I alleles: NetMHCpan4.2^23^, NetMHCpan4.1^24^, NetMHCpan4.0^11^, ANN4.0^25^, SMMPMBEC 1.0^26^, SMM1.0^27^, PickPocket1.1^28^, netMHCcons1.1^29^ and MHCflurry2.0^12^. The majority of these algorithms are trained as pan-allele models, allowing them to generate predictions for mouse alleles even when human data were the primary training focus, with ANN and SMM or SMMPMBEC being exceptions using only data available for the specific allele. Most methods rely on neural networks trained on pMHC binding affinity data and MHC elution mass spectrometry datasets. NetMHCons is a consensus model that integrates predictions from NetMHCpan, ANN and PickPocket to improve overall performance. These algorithms predict the binding affinity, or KD, of peptides for their cognate MHC-I molecule (Fig. 1A) reported as an IC50, or the concentration of peptide required to bind 50% of the available MHC molecules. Our neoantigens were assigned a wide range of predicted affinities by these algorithms, ranging from 1.83 nM to 6469 nM. Generally, neoantigens with a binding affinity of <500 nM are considered unlikely to elicit a T cell response, while a binding affinity of <50 nM predicts strong immunogenicity^8^. When applying this criteria, the majority of programs classified most of the neoantigens as immunogenic, with the notable exception of PickPocket1.0, which classified only 60% of the neoantigens below 500 nM and 13% below 50 nM. MHCflurry notably classified 80% of the neoantigens as strong (<50 nM), substantially more than the next highest predictor, NetPanMHC4.1, at 60%. All of these neoantigens have been demonstrated to elicit a CD8+ T cell response *in vivo* highlighting the predictive limitations of these models in our dataset.

The algorithms generally ranked the neoantigens in a similar order with high Spearman correlation coefficients across both alleles tested (Fig. 1B). PickPocket 1.1 was by far the most distinct algorithm, showing little correlation with the other models, likely driven by its consistently weaker MHC affinity predictions. MHCflurry was also less correlated with the other algorithms and generally predicted higher affinity antigens. When using the Pearson correlation coefficient to compare the linear relationship of binding affinities across the algorithms, the outputs generally grouped based on the underlying training data for the algorithms, with the pan-allele algorithms grouping separately from the allele-specific algorithms and netMHCcons showing a heavy influence from NetPanMHC one of the algorithms it uses for it consensus score (Fig. 1C).

### In Vitro Affinity Measurements Poorly Correlate with In Silico Values

**Figure 2.**
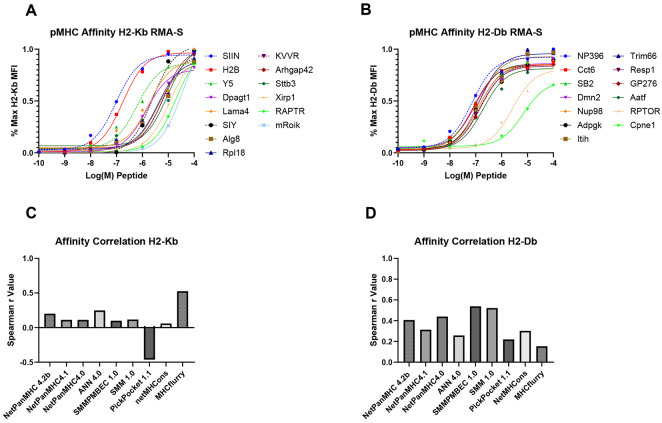
In Vitro Affinity Measurements Poorly Correlate with In Silico Values (A-B) Relative peptide-MHC (pMHC) affinity (K_D_) for each neoantigen as assessed by flow cytometry staining for stabilized MHC-I molecules, H-2K^b^ (A) and H-2D^b^ (B), on RMA-S cells incubated with exogenous peptide. The plotted curve uses a S4PL model with a Hill Slope of 1. (C-D) Plotted Spearman correlation coefficients for experimentally determined (RMA-S assay) versus *in silico* predicted IC50 values for H2-K^b^ (C) and H2-D^b^ (D).

To determine how predicted binding affinities align with experimentally determined values, we employed an RMA-S peptide affintiy assay^30^, which is commonly used to experimentally determine the pMHC equilibrium dissociation constant (K_D_). RMA-S cells that contain empty MHC I molecules are incubated with exogenous peptide, which stabilizes MHC molecules on the surface and a binding curve can be generated to determine relative K_D_. Performing this assay on our library of neoantigens, we observed a range of K_D_ values spanning several orders of magnitude, but generally higher than the computationally predicted values (Fig. 2A-2B). It is important to note that the RMA-S stabilization assay provides a relative measure of pMHC binding rather than an absolute affinity, as stabilization is inferred from MHC levels at the cell surface rather than an interaction solely between the MHC molecule and peptide. In addition, because MHC stabilization is detected using allele-specific antibodies with distinct binding properties, measurements are most appropriately compared within a given allele. For the neoantigens binding the H2-K^b^ allele, the strongest binder was SIINFEKL, which is the mostly commonly used model neoantigen across mouse tumor studies, followed closely by the tumor-derived neoantigen H2bc15. The other model neoantigens exhibited K_D_ values within the range of tumor-derived neoantigens. Neoantigens binding the H2-D^b^ allele displayed a more compact range of K_D_ values, with immunodominant LCMV-derived NP396 narrowly assuming the top position. Only two neoantigens exhibited a log-fold weaker K_D_ than the top antigen, and both were predicted by the majority of the *in silico* algorithms to be among the weaker binders in the library. However, notably, the strongest binder, NP396, was also predicted to be among the weakest binders by most of the *in silico* algorithms.

In general, there was poor agreement between the ranking of neoantigen affinities by *the in silico* models and the experimentally measured K_D_ values, with low Spearman correlation coefficients (Fig. 2C-2D). The models performed better at ranking neoantigens that bind the H2-D^b^ allele than the H2-Kb allele; however, the strongest correlation coefficient was ~0.5. Ultimately, these findings suggest a key limitation of *in silico* prediction programs: while they can generally predict good MHC binders and approximate binding affinity, they poorly predict the ranking of MHC binding affinities among strong neoantigens.

### pMHC Stability Poorly Correlates with Affinity

**Figure 3.**
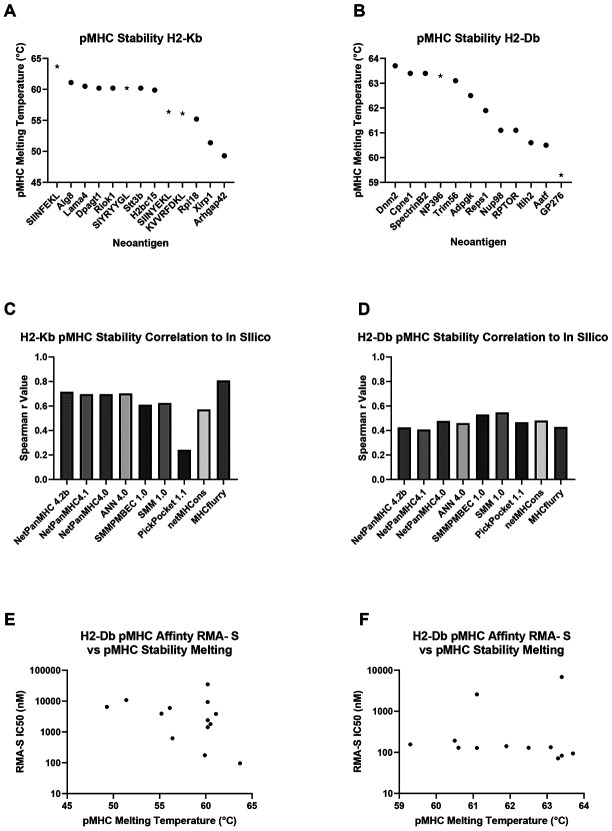
pMHC Stability Poorly Correlates with Affinity (A-B) Relative pMHC stability (K_off_) for each neoantigen as measured by differential scanning fluorimetry of pMHC H-2K^b^ (A) and H-2D^b^ (B) complex thermal melting. Tumor-derived neoantigens, circles; model neoantigens, stars. Melting temperature of the complex is defined as the minimum value of the inverse derivative of florescence over time (−dF/dT). (C-D) Plotted Spearman correlation coefficients for experimentally determined stability versus *in silico* predicted IC50 values for H2-K^b^ (C) and H2-D^b^ (D). (E) RMA-S assay pMHC affinity IC50 values plotted against the pMHC stability melting temperature values for each neoantigen for the H2-K^b^ (E) and H2-D^b^ (F) alleles.

Another measurement of MHC binding strength is K_off_, or stability, which reflects the dissociation rate of the pMHC complex and is related to K_D_ as K_D_ = K_off_/K_on_. The melting temperature of pMHC is inversely related to K_off_, where a higher melting temperature is equivalent to a longer half-life of the pMHC complex^31^. Multiple studies suggest this measurement can better predict the immunogenicity of an antigen than K_D_ in both viral and tumor models^7,9,32-34^. We used UV-exchangeable MHC monomers to generate pMHC complexes for our neoantigen library and generated pMHC melting curves by differential scanning fluorimetry. In line with our pMHC affinity measurements, SIINFEKL had the highest melting temperature (indicative of a lower K_off_) than the other H2-K^b^ neoantigens (Fig. 3A-3B). Again, neoantigens binding the H2-K^b^ allele displayed a wider range of values compared to H2-D^b^, with greater representation of weaker binders. Melting temperature values correlated, on average, better with *in silico* predicted rather than experimentally measured pMHC binding affinity, which was somewhat surprising given the *in silico* algorithms predict K_D_ and not K_off_ (Fig. 3C-3D). However, *in silico* algorithms that were trained in part by eluted peptides in theory should overrepresent peptides that are more stable. Notably, MHCflurry showed the highest correlation with both the experimentally measured pMHC stability and affinity for the H2-K^b^ allele, while PickPocket performed the worst. In contrast, for the H2-D^b^ allele, there was no clear top-performing *in silico* algorithm for predicting stability.

When comparing the ranked order of neoantigens measured by the affinity and stability assays, greater agreement was observed for neoantigens binding the H2-K^b^ allele than H2-D^b^. A narrower range of melting temperatures for H2-D^b^ binders may have contributed, spanning only 4.4°C compared to a 14.4°C for H2-K^b^ binders, and melting temperatures notably failed to identify the two outlier H2-D^b^ neoantigens in the RMA-S assay. In contrast, neoantigens binding H2-K^b^ showed more agreement between the stability and affinity measurements, and in cases where disagreement was observed, the neoantigens exhibited higher melting temperatures than would be predicted by their K_D_ values. Notably, no neoantigens displayed both low stability and high affinity, or vice versa, suggesting the measurements are concordant at a high level (Fig. 3E-3F).

### pMHC Stability Correlates with In Vivo Immunogenicity

**Figure 4.**
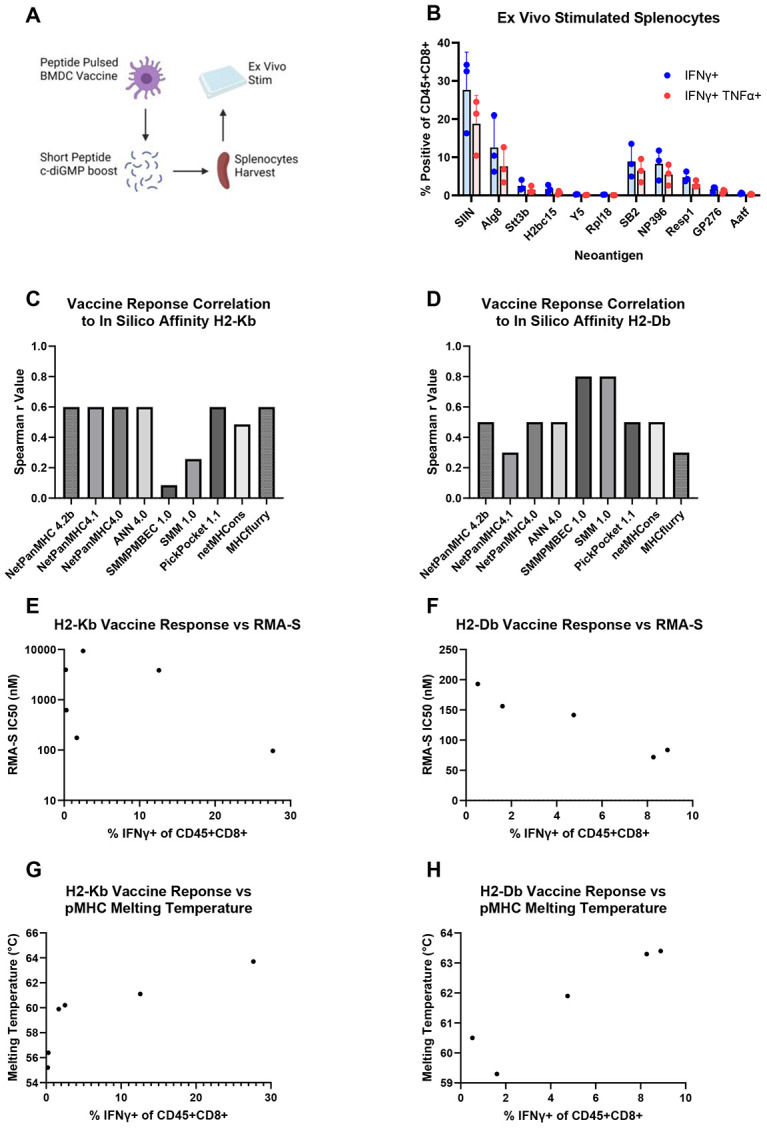
pMHC Stability Correlates with In Vivo Immunogenicity (A) Schematic of neoantigen peptide-pulsed bone marrow-derived dendritic cell vaccine (1.5 million cells) delivered into wild-type C57BL/6 mice with cyclic di-GMP adjuvant (25 μg) followed by a neoantigen peptide (6 μg) and adjuvant boost one week later. (B) The percent of IFNγ+ or IFNγ+ TNFα+ CD8+ T cells is graphed for each neoantigen-targeted vaccine. (C-D) The plotted Spearman correlation coefficient for the percent IFNγ+ cells for each neoantigen compared to the predicted IC50 values from each *in silico* algorithm for the H2-K^b^ (C) and H2-D^b^ (D) alleles. (E-F) RMA-S assay pMHC affinity IC50 values plotted against the percent IFNγ+ cells for each neoantigen for the H2-K^b^ (E) and H2-D^b^ (F) alleles. (G-H) Plot of pMHC stability melting temperature values against the percent IFNγ+ cells for each neoantigen for the H2-K^b^ (G) and H2-D^b^ (H) alleles.

To determine which measurements of pMHC binding strength best correlate with *in vivo* neoantigen immunogenicity, we selected a subset of neoantigens spanning the full range of predicted and experimentally measured values of affinity and stability. Wild-type mice were vaccinated with bone marrow-derived dendritic cells pulsed with the respective neoantigen short-peptides, followed by a booster one week later consisting of a short-peptide vaccine formulated with a cyclic di-GMP adjuvant (Fig. 4A). The goal of our vaccine strategy was to elicit a below saturating response to achieve a range of responses for the neoantigen set, and we observed detectable responses for the majority of neoantigens (3 of 5 for H2-K^b^ and 4 of 5 for H2-D^b^; Fig. 4B). Overall, the *in silico* programs poorly predicted the rank order of the CD8+ T cell response to each neoantigen (Fig. 4C-4D). In contrast, pMHC stability successfully ranked the neoantigens binding H2-K^b^ and nearly recapitulated the ranking for H2-D^b^ neoantigens (R^2^ values of 0.77 and 0.88, respectively; Fig. 4E-4F). The affinity assay’s ability to predict the immunogenicity of the neoantigens was allele dependent; the rank order was poorly correlated for H2-K^b^, but highly correlated for H2-D^b^ with an R^2^ value of 0.94 (Fig. 4G-4H). These results demonstrate that *in silico* prediction algorithms did not reliably stratify neoantigen immunogenicity for our neoantigen library, whereas *in vitro* measurements of pMHC binding strength, especially stability, were more accurate predictors of *in vivo* neoantigen immunogenicity.

### pMHC Stability Measurement to Benchmark Immunogenicity of Novel Neoantigens

**Figure 5.**
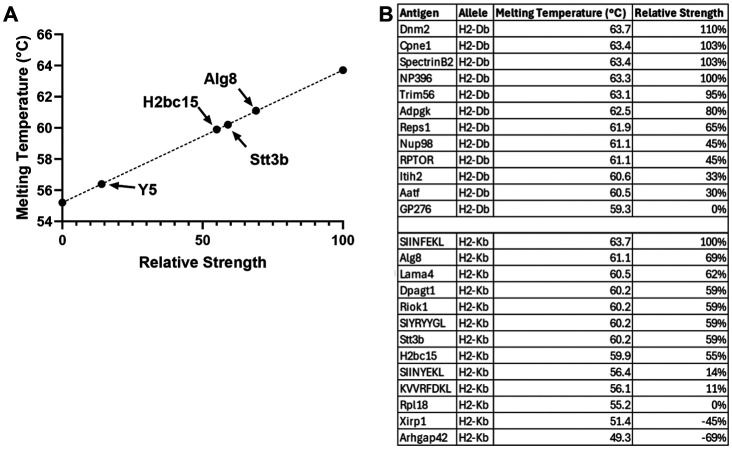
pMHC Stability Measurement to Benchmark Immunogenicity of Novel Neoantigens (A) Plot of the melting temperatures of Sttb3, H2bc15, Alg8 and SIINYEKL using SIINFEKL and Rpl18 as benchmarks to compare the relative strength of neoantigens. (B) Table of relative neoantigen strength calculated as a percentage of the strongest reference neoantigen.

Our observations align with publications demonstrating stability to be a better measurement of immunogenicity than affinity^7,9,32-34^. The melting temperature assay we used to measure stability can be easily replicated and may help investigators better predict the relative immunogenicity of neoantigens in their systems. MHC-I monomers are currently available to academic investigators through the NIH Tetramer Core free of charge and the melting temperature assay can be performed with a standard real-time PCR machine, making measurement of pMHC stability a simple and low-cost method for predicting neoantigen immunogenicity. We further propose benchmarking novel neoantigens against a known strong and weak neoantigen from our library as standards to facilitate comparison of neoantigen immunogenicity across labs and reagent lots. Specifically, we propose using SIINFEKL and Rpl18 as the pair for H2-K^b^ and NP396 and GP276 as the pair for H2-D^b^. By measuring the melting temperature of these two standards, one can determine the strength of an unknown on a fixed scale with SIINFEKL being 100% strength and Rpl18 being the baseline. Here, as an example, we plotted the melting temperature of Stt3b and see that it is 59% the strength of SIINFEKL; this was closest to and just above H2bc15 at 55%, which would be predicted to have similar immunogenicity and indeed gave a similar response in our vaccine study (Fig. 5A-5B, compared to Fig. 4B). We further plotted Alg8, which was 69% the strength of SIINFEKL and SIINYEKL which was 14% the strength of SIINFEKL and corresponding generated a larger and small vaccine response respectively compared to Stt3b and H2bc15 (Fig. 5A-5B, compared to Fig. 4B). By comparing any new value obtained to our reference table one can quickly gauge the relative strength of a new neoantigen (Fig. 5B).

## DISCUSSION

A persistent challenge in the preclinical study of tumor neoantigens is the lack of a reliable way to compare neoantigen strength across tumor models; this makes it difficult to compare results across studies, as anti-tumor T cell responses are shaped by both tumor-intrinsic factors and neoantigen-intrinsic properties. In this work, we have shown that a simple *in vitro* approach of measuring pMHC stability can benchmark relative neoantigen immunogenicity and provide an important framework for comparing neoantigen-driven phenotypes across studies.

This study evaluated the extent to which *in vitro* measurements and *in silico* predictions of pMHC binding strength could stratify immunogenicity of a library of known mouse tumor and model neoantigens. We found that *in silico* programs poorly ranked the neoantigens in order of *in vivo* immunogenicity. This may be because these algorithms were not trained to discern the difference in strength between immunogenic antigens, but rather to select the peptides that are likely to bind MHC out of a much larger set of non-binders. Success is measured from correct classification of peptides into the binary categories of binder or non-binder. In line with this, a meta-analysis of peptides with multiple MHC affinity measurements from the Immune Epitope Database looking for agreement between different experimental methods showed strong agreement using a binder/non-binder classification. Yet, the subset of antigens with the highest binding affinities poorly correlated across the different measured affinity values failing to rank the peptides in a similar order with a correlation coefficient of ~0.2^35^. This data suggest *in silico* prediction algorithms will also have a difficult time ordering high affinity antigens correctly. To assess the relative predictive power of experimentally measured pMHC affinity, we employed the commonly used RMA-S cell MHC binding affinity assay. While this method ranked the immunogenicity of H-2D^b^-restricted neoantigens with relative accuracy, H-2K^b^ rankings were poorly correlated with both *in silico* predictions and *in vivo* immunogenicity. Notably, the H-2D^b^ neoantigen set was collectively skewed toward higher affinity MHC binders, which could suggest that the RMA-S assay more accurately ranks strong neoantigens. Altogether, however, experimentally measured affinity was a surprisingly poor predictor of immunogenicity.

A growing body of research suggests that pMHC stability is more predictive of antigen immunogenicity than pMHC affinity. In a study comparing immune responses to viral antigens, pairs of antigens with the same affinity were found to generate sizably different CD8+ T cell responses; the difference was attributed to differential stability, where the more stable antigen consistently garnered the largest immune response^9^. In tumor models, it has similarly been observed that the relative stability, rather than affinity, of neoantigens can better predict the relative magnitude of neoantigen-specific CD8+ T cell responses^7^. This also appears to hold true in the context of therapeutic vaccination, where an mRNA-lipid nanoparticle vaccine concurrently expressing multiple neoantigens elicited the largest CD8+ T cell responses against the highest stability neoantigens^36^. Consistent with these observations, experimental measurement of relative pMHC stability by differential scanning fluorimetry accurately predicted the relative *in vivo* immunogenicity of our library of neoantigens. Therefore, we propose that pMHC stability is a reliable metric to predict the relative strength of neoantigens and have provided a framework for benchmarking newly identified mouse neoantigens against our reference set using a simple pMHC melting temperature-based assay.

In the absence of well-characterized tumor-derived neoantigens, many studies have relied on model neoantigens, such as SIINFEKL and others included here, as surrogates to evaluate tumor neoantigen-specific T cell responses. Model neoantigens offer several advantages, including decades of use across multiple tumor models and the wide availability of experimental tools for tracking neoantigen-specific T cell responses, including pMHC tetramers and T cell receptor (TCR) transgenic mice. However, it has been theorized that immunoediting may bias tumors toward a repertoire of weaker antigens, potentially limiting the relevance of model neoantigens for preclinical study. In our study, we did not observe a broad distinction between tumor-derived neoantigens and commonly used model neoantigens in terms of pMHC binding strength or *in vivo* vaccine response. This is consistent with several studies showing that tumors can harbor mutations with very strong neoantigens, including with predicted pMHC binding strength equivalent to SIINFEKL. Notably, two recent studies in genetically engineered mouse models of lung and pancreatic cancer observed that CD8+ T cell responses against SIINFEKL and the tumor neoantigens Alg8 and Lama4 were of comparable magnitude and phenotype^7,37^. In our dataset, Alg8 and Lama4 ranked near SIINFEKL in relative pMHC stability; these findings might suggest there is a threshold of pMHC stability beyond which CD8+ T cell responses become maximal in the tumor setting.

Despite the strong predictive value of pMHC binding strength, this framework has limitations. While pMHC stability is a major determinant of surface pMHC density, baseline expression of the source protein also influences antigen presentation. For example, we found that the neoantigen Rpl18 has relatively low pMHC affinity and stability. However, it was identified as an immunodominant neoantigen in the MC38 colon cancer model, which may be attributable to its high expression levels as a ribosomal protein^18^. Notably, in a second study characterizing the immunogenicity of MC38 neoantigens, Rpl18 was not identified as highly immunogenic^17^. Differences in neoantigen clonality between two differentially maintained MC38 lines could potentially have resulted in variable abundance of Rpl18 and explain the difference in immunogenicity. Additional factors have been recognized to contribute to immunogenicity, including proteasomal processing^38-40^, naïve T cell precursor repertoire and TCR affinity for pMHC^41^. However, across studies, peptide-MHC binding strength is generally the strongest predictor with the least variance across individuals^42^.

The ability to determine the quality of neoantigens is becoming increasingly relevant as neoantigen prediction moves from preclinical studies into the clinic with the development of personalized cancer vaccines. In this therapeutic approach, tumor DNA mutations are identified by exome sequencing, candidate neoantigens are predicted using *in silico* algorithms that consider RNA expression of proteins, and top predicted targets are manufactured into pooled mRNA or peptide-based vaccines. However, studies of endogenous neoantigen responses consistently show that the number of predicted neoantigens far exceeds the number that elicit detectable T cell responses, and that the magnitude of these responses varies dramatically, ranging from only a few cells to thousands^2-4,43,44^. This narrowing of immune responses from predicted to observed is also true in neoantigen vaccine trials where often tens of neoantigens are vaccinated against but very few generate a detectable response^45-49^. Clearly, there is room to improve how we select neoantigens to incorporate into these vaccines. Our observations here suggest that future vaccine design may benefit from incorporating measured or predicted stability of pMHC complexes. While directly measuring pMHC stability may be challenging to incorporate into the clinical vaccine pipeline, it should be possible to generate a large reference dataset of neoantigen stability measurements to train the next generation of prediction models for neoantigen selection. Ultimately, predicting the immunogenicity of peptides remains a difficult task, but we have found that peptide stability is the best correlation with *in vivo* immunogenicity among a set of mouse neoantigens.

## METHODS

### RMA-S Cell MHC Affinity Assay

RMA-S cells were cultured in RPMI 1640 media (10% fetal bovine serum (FBS), 2 mM L-glutamine, 10 mM HEPES, 1X NEAA, 1X β-mercaptoethanol and 50 U/uL Penicillin/Streptomyocin) at 37°C at 5% CO_2_ and transferred to 26°C at 5% CO_2_ for 4 hours to stabilize MHC molecules on the cell surface. Cells were then plated at 100,000 cells per well in 96-well flat bottom plates with 10-fold serial dilutions of the respective peptide for 1 hour at 26°C. Cells were moved to 37°C for one hour to internalize empty MHC molecules and then kept on ice to stop MHC trafficking. Cells were washed with FACS buffer (1% heat-inactivated FBS in PBS) and stained with either H2-K^b^ or H2-D^b^ antibody (Cat. No.17-5958-82, Cat. No.16-5999-82) as appropriate for the peptide for 15 minutes on ice, then fixed with 4% paraformaldehyde (PFA) for 15 minutes on ice. Cells were analyzed on a BD FACSymphony^™^ A5 SE Cell Analyzer.

Mean fluorescence intensity (MFI) values were normalized to the baseline and highest value within an experiment. Values were then plotted in GraphPad Prism and a S4PL equation with a Hill Slope of 1 was used to fit the data and generate IC50 values.

### Differential Scanning Fluorimetry Assay for pMHC Thermal Melting

MHC I monomers (NIH Tetramer Core) loaded with the UV-cleavable peptide FAPGNYJAL were combined with the exchange peptide in PBS at a concentration of 0.2 mg/mL monomer and 0.4 μM peptide in a 96 well plate. The solution was incubated for 1 hour in a UVA light box. Loaded monomers were centrifuged at 3300g for 5 minutes and supernatant transferred to a final tube. 18 μL of this solution was combined with 2 μL of 10X SYPRO Orange (Invitrogen, Cat. No. S6650) in a 96 well plate and incubated for 10 minutes to allow for the dye to bind the protein. The plate was assayed on a QuantStudio 7 Flex Real-Time PCR System; samples were equilibrated at 25°C for 1 minute then heated at a rate of 0.05°C/s from 25°C to 95°C with continuous fluorescence measurements at an excitation of 470nm and emission of 570nm. Melting curves were normalized to the minimum and maximum fluorescence value and the inverse of the first derivative (−dF/dT) was then calculated. The melting temperature is defined as the minimum value of −dF/dT.

### Bone Marrow-Derived Dendritic Cell Vaccination

Bone marrow cells were collected by euthanizing mice and removing the femur and tibia. The heads of the bones were clipped and placed facing down into a 0.5-mL tube nested in a 1.5-mL microcentrifuge tube. These tubes were flash spun at maximum speed in a microcentrifuge to extract the bone marrow. Red blood cells were lysed with 1X Red Blood Cell (RBC) Lysis Buffer (eBioscience; Cat. No. 00-4333-57). Cells were filtered through 70 μm cell strainers, counted and resuspended in Dendritic Cell Media (DCC) (DMEM, 10% FBS, 2 mM L-glutamine, 50 U/uL Pen/strep, 1X β-mercaptoethanol, 100 ng/mL Flt-3L and 3.75 ng/mL GM-CSF) at 1x10^6^ cells/mL. 1x10^7^ cells were plated per well in a 6 well plate and cultured at 37°C. On day 4, each well was supplemented with additional Flt-3L 1 μg, GM-CSF 37.5 ng and 0.5X β-mercaptoethanol. On day 7, the media and cells were collected, spun down at 1200 rpm for 5 minutes, resuspended in fresh DCC and replated 1x10^6^ cells/mL in a 6 well plate. On day 9, CpG (1 ug/mL) was added to generate mature bone marrow-derived dendritic cells (BMDCs). On day 10, the BMDCs were collected and resuspended in 10 mL of DCC media with 1 μg/mL of the appropriate neoantigen short-peptide in 15-mL conical tubes. These tubes were incubated with a loose cap in a 37°C incubator at 5% CO_2_ for 2 hours. The neoantigen-pulsed BMDCs were then washed with PBS and resuspended at 1.5x10^7^ cells/mL and 1.5 million cells were injected in 100 μL volume subcutaneously at the tail base. One-week later, mice were boosted with 6 μg of neoantigen short-peptide and 25 μg of cyclic di-GMP adjuvant in PBS and injected in 100 μL volume subcutaneously at the tail base. Spleens were harvested 1 week after the booster dose.

Spleens were mashed through a 70 μm filter, spun down at 1200 rpm for 5 minutes, then incubated in 1X RBC Lysis Buffer for 5 minutes on ice. Pellets were then resuspended in R10 media (RPMI 1640, 10% FBS, 2 mM L-glutamine, 10 mM HEPES, 1X NEAA, 1X β-mercaptoethanol and 50U/μL Penicillin/streptomycin). 1.5 million splenocytes were plated in R10 for 5 hours with 1X Brefeldin-A and 1 μg/mL of the corresponding neoantigen short-peptide. Cells were then washed and stained with Live/Dead Fixable Dead Cell Stain (Invitrogen, Cat. No. L34957) for 30 minutes on ice, then anti-mouse CD45 (Invitrogen, Cat. No. 47-0451-82) and CD8α (BD Biosciences, Cat. No. 553031) antibodies for 15 minutes on ice. Cells were fixed for 1 hour at room temperature and permeabilized with the eBioscience Intracellular Fixation and Permeabilization Buffer Set (Invitrogen, Cat. No. 88-8824-00). Cells were then stained overnight with anti-mouse IFNγ (BioLegend, Cat. No. 505822) and TNFα (BioLegend, Cat. No. 506306) antibodies. Cells were analyzed on a BD FACSymphony^™^ A5 SE Cell Analyzer.
